# Oral Factor XIa Inhibitors for Secondary Prevention After Noncardioembolic Ischemic Stroke or High‐Risk Transient Ischemic Attack: Systematic Review and Meta‐Analysis

**DOI:** 10.1002/brb3.71846

**Published:** 2026-10-22

**Authors:** Mirza Ammar Arshad, Hina Nadeem, Maham Afzal, Abbas Ali, Rasheeda Shehroz, Affan Khalid, Zaiya Waseem, Ahsan Raza Raja, Eman Naeem Cheema, F. N. U. Hizbullah, Gulleena Junaid, Asad Ali Ahmed Cheema

**Affiliations:** ^1^ Department of Medicine Rahbar Medical & Dental College Lahore Pakistan; ^2^ Department of Medicine Jinnah Sindh Medical University Karachi Pakistan; ^3^ Department of Medicine Fatima Jinnah Medical University Lahore Pakistan; ^4^ Department of Medicine Gomal Medical College Dera Ismail Khan Pakistan; ^5^ Department of Medicine Liaquat University of Medical & Health Sciences Jamshoro Pakistan; ^6^ Department of Medicine Lahore Medical & Dental College Lahore Pakistan; ^7^ Department of Internal Medicine AtlantiCare Regional Medical Center New Jersey USA; ^8^ Department of Medicine Aga Khan University Karachi Pakistan; ^9^ Department of Medicine CMH Lahore Medical College & Institute of Dentistry Lahore Pakistan; ^10^ Department of Medicine Chandka Medical College Larkana Pakistan; ^11^ Department of Medicine HBS Medical and Dental College Islamabad Pakistan; ^12^ Department of Medicine, International School of Medicine International University Kyrgyzstan Bishkek Kyrgyzstan

**Keywords:** factor XIa inhibitors, ischemic stroke, transient ischemic attack, secondary prevention, antiplatelet therapy

## Abstract

**Introduction:**

Patients with recent noncardioembolic ischemic stroke or high‐risk transient ischemic attack (TIA) remain at risk of recurrent events despite antiplatelet therapy. Oral factor XIa (FXIa) inhibitors may reduce thrombotic risk while limiting bleeding, but their adjunctive efficacy and safety require clarification.

**Methods:**

PubMed, Embase, Cochrane Library/CENTRAL, and ClinicalTrials.gov were searched through May 2026 for randomized, double‐blind, placebo‐controlled trials of oral FXIa inhibitors added to antiplatelet therapy. Primary efficacy and safety outcomes were recurrent ischemic stroke and major bleeding. Risk ratios (RRs) with 95% confidence intervals (CIs) were pooled using random‐effects models.

**Results:**

Three trials randomized 16,501 participants overall, of whom 14,239 contributed to the primary selected‐dose efficacy analysis. FXIa inhibition reduced recurrent ischemic stroke (413/6927 vs. 579/7312; RR 0.74, 95% CI 0.65–0.84; I^2^ = 0%; *p* < 0.00001). No statistically significant differences were observed in major bleeding (RR 1.12, 95% CI 0.87–1.44), major or clinically relevant non‐major bleeding (RR 1.40, 95% CI 0.91–2.15), clinically relevant non‐major bleeding (RR 1.49, 95% CI 0.85–2.60), minor or non‐actionable bleeding (RR 0.94, 95% CI 0.84–1.05), intracranial or intracerebral hemorrhage (RR 1.16, 95% CI 0.75–1.79), or fatal bleeding (RR 1.75, 95% CI 0.74–4.17); however, confidence intervals for several bleeding outcomes were wide and did not exclude clinically important harm. Findings remained consistent in the all‐dose sensitivity analysis (RR 0.77, 95% CI 0.68–0.86; I^2^ = 0%).

**Conclusions:**

In patients with recent noncardioembolic ischemic stroke or high‐risk TIA receiving antiplatelet therapy, pooled randomized evidence showed a reduction in recurrent ischemic stroke with oral FXIa inhibition; however, the estimate was predominantly driven by the phase 3 asundexian trial, limiting certainty regarding generalization across individual FXIa inhibitors. Major bleeding and other safety outcomes were not significantly increased, although rare‐event estimates were imprecise and clinically important harm cannot be excluded.

## Graphical abstract

Oral factor XIa inhibitors, added to background antiplatelet therapy after noncardioembolic ischemic stroke or high‐risk TIA, were associated with a reduction in recurrent ischemic stroke in pooled randomized evidence. Major bleeding and other safety outcomes were not significantly increased, although rare‐event estimates remained imprecise and clinically important harm could not be excluded.

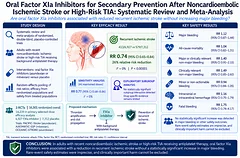

## 1 Introduction

Patients with noncardioembolic ischemic stroke and high‐risk transient ischemic attack (TIA) are at significant risk for subsequent stroke and TIA, especially in the first few months after the initial stroke or TIA. For patients in whom cardiac embolism is not a known indication for anticoagulation (such as those without any atrial fibrillation or other known cardioembolic disease), antiplatelet therapy continues to be the recommended antithrombotic therapy for secondary prevention (Kleindorfer et al. 2021; Dawson et al. 2021). Clopidogrel or ticagrelor plus aspirin yield a lower rate of early recurrent ischemic events in selected patients with minor ischemic stroke or high‐risk TIA (Wang et al. 2013; Johnston et al. 2018, 2020). This benefit comes with a higher risk of bleeding, though, and there is no justification for intensifying antiplatelet therapy for extended periods or indiscriminately. Despite current guideline‐directed therapy, therefore, there remains a significant residual risk of ischemic stroke recurrence, a risk that warrants therapeutic interventions with higher efficacy to reduce the risk of stroke recurrence, but with fewer hemorrhagic complications.

Factor XI (FXI), particularly its activated form factor XIa (FXIa), is a biologically attractive target for antithrombotic therapy. The role of FXI in hemostasis is primarily involved in thrombin amplification, propagation and stabilization of pathologic thrombi, but seems to play a less critical role in normal hemostasis than thrombin or FXa (Hsu et al. 2021). People with severe congenital FXI deficiency are less likely to develop ischemic stroke and research in humans has also linked lower FXI activity with a lowered thrombotic risk (Salomon et al. 2008; Georgi et al. 2019). Importantly, a relatively moderate spontaneous bleeding phenotype in the absence of FXI deficiency exists, but bleeding can occur after trauma or surgery. These observations have led to the idea that partial inhibition of FXIa could uncouple the processes of thrombosis from hemostasis (Hsu et al. 2021). As a result, small‐molecule FXIa inhibitors such as asundexian and milvexian have been developed and are being investigated as an adjunct to antiplatelet therapy following noncardioembolic ischemic stroke or high‐risk TIA. Nevertheless, their addition to single or dual antiplatelet therapy could still increase clinically relevant or major bleeding, making assessment of the overall efficacy–safety balance essential.

Randomized evidence has progressed from a phase 2 dose‐finding study to a clinical‐outcome evaluation stage 3 study. Asundexian failed to significantly decrease the primary composite end point of recurrent symptomatic ischemic stroke or covert brain infarction detected by magnetic resonance imaging (MRI), although the estimates for the 50 mg dose were numerically in favor of this dose (Shoamanesh et al. 2022).

Similarly, the phase 2 AXIOMATIC‐SSP did not demonstrate a significant dose–response effect of milvexian on its composite cerebral ischemic endpoint but provided further efficacy and safety data across multiple doses (Sharma et al. 2024). More recently, the phase 3 OCEANIC‐STROKE showed that asundexian 50 mg once daily, taken in addition to protocol‐specified antiplatelet therapy, decreased ischemic stroke events when compared to placebo, without a significant increase in major bleeding events (Sharma et al. 2026). Previous meta‐analyses either predated the current phase 3 evidence or combined heterogeneous primary‐ and secondary‐prevention populations, clinical indications, FXI/FXIa inhibitors, and comparators such as placebo and direct oral anticoagulants (Palaiodimou et al. 2023; Zahid et al. 2025). The effectiveness and safety of oral FXIa inhibition specifically as a therapeutic option as an adjunct to antiplatelet therapy after the onset of a noncardioembolic ischemic stroke or a high‐risk TIA has not been adequately defined with the full phase 2 and phase 3 randomized evidence.

Therefore, we conducted a systematic review and meta‐analysis of randomized, double‐blind, placebo‐controlled trials comparing oral FXIa inhibitors plus protocol‐specified background antiplatelet therapy with matching placebo plus the same antiplatelet regimen in adults with recent noncardioembolic ischemic stroke or high‐risk TIA. The primary efficacy outcome was recurrent ischemic stroke, and the primary safety outcome was major bleeding. Secondary outcomes included all‐cause mortality, major or clinically relevant non‐major bleeding, clinically relevant non‐major bleeding alone, minor or non‐actionable bleeding, intracranial or intracerebral hemorrhage, and fatal bleeding. We also performed a sensitivity analysis incorporating all eligible nonterminated active‐dose groups from the phase 2 trials to examine the robustness of the primary efficacy finding.

## 2 Methods

### 2.1 Reporting Framework and Protocol Registration

This systematic review and meta‐analysis were conducted and reported in accordance with the Preferred Reporting Items for Systematic Reviews and Meta‐Analyses (PRISMA) 2020 statement (Page et al. 2021). The review protocol was registered in the International Prospective Register of Systematic Reviews (PROSPERO; CRD420261439705) before data extraction and quantitative synthesis (Booth et al. 2012). The eligibility criteria, outcomes, and analytic strategy were defined according to a prespecified population, intervention, comparator, outcomes, and study‐design framework. Ethics approval and individual informed consent were not required because this study used aggregate data from previously published trials.

### 2.2 Eligibility Criteria

Randomized, double‐blind, placebo‐controlled trials were eligible if they enrolled adults with acute noncardioembolic ischemic stroke or high‐risk TIA; evaluated an oral FXIa inhibitor added to protocol‐specified background antiplatelet therapy; used a matching placebo with the same background antiplatelet regimen; and reported recurrent ischemic stroke or bleeding outcomes. Trials evaluating asundexian, milvexian, or another oral FXIa inhibitor were eligible regardless of trial phase, geographic location, or duration of follow‐up. Studies were excluded if they enrolled populations with atrial fibrillation, cardioembolic stroke, acute coronary syndrome, venous thromboembolism, orthopedic surgery, or other clinical conditions outside the target population of recent noncardioembolic ischemic stroke or high‐risk TIA. We also excluded nonrandomized studies, case reports, reviews, editorials, and studies that did not provide extractable outcome data.

In multiarm phase 2 dose‐ranging trials, one active‐treatment arm was included in the primary analysis to prevent the repeated analysis of the same placebo arm. All eligible doses of oral FXIa inhibitors could be included in the trial, and the registered PROSPERO protocol did not stipulate that a particular dose from multiarm trials be selected. The regimen subsequently progressed to phase 3 development for secondary stroke prevention (asundexian 50 mg daily in the phase 2b PACIFIC‐Stroke and milvexian 25 mg twice daily in AXIOMATIC‐SSP [Shoamanesh et al. 2022; Sharma et al. 2024]) was therefore used for the primary analysis. Given the possibility that this dose‐selection approach may have influenced the pooled estimate, this analysis was repeated as an all‐dose sensitivity analysis, which included all dose groups that were maintained and not terminated during the phase 2 trials and the same pooled placebo group only once.

### 2.3 Information Sources and Search Strategy

The following databases or registries were searched from database/registry inception to May 2026: PubMed, Embase, Cochrane Central Register of Controlled Trials (CENTRAL), and ClinicalTrials.gov. There were no language or publication date limitations. The search strategies included controlled vocabulary and free‐text terms to identify ischemic stroke or TIA along with terms related to FXI/FXIa inhibition and individual agents, such as asundexian and milvexian. The verbatim search strategy for each source is included, along with the database/platform, controlled vocabulary, field tags, Boolean operators, search date, restrictions, and number of records found (Table S1A and Table S1B).

### 2.4 Study Selection

All retrieved records were independently screened by two reviewers (FH, GJ) by reading the titles, abstracts, and trial‐register records. The reviewers were also independent full text assessors of potentially eligible reports. Disagreements were cleared up by discussion and, if necessary, with a third reviewer (MAA). The study‐selection process is presented in the PRISMA flow diagram (Figure 1).

**FIGURE 1:**
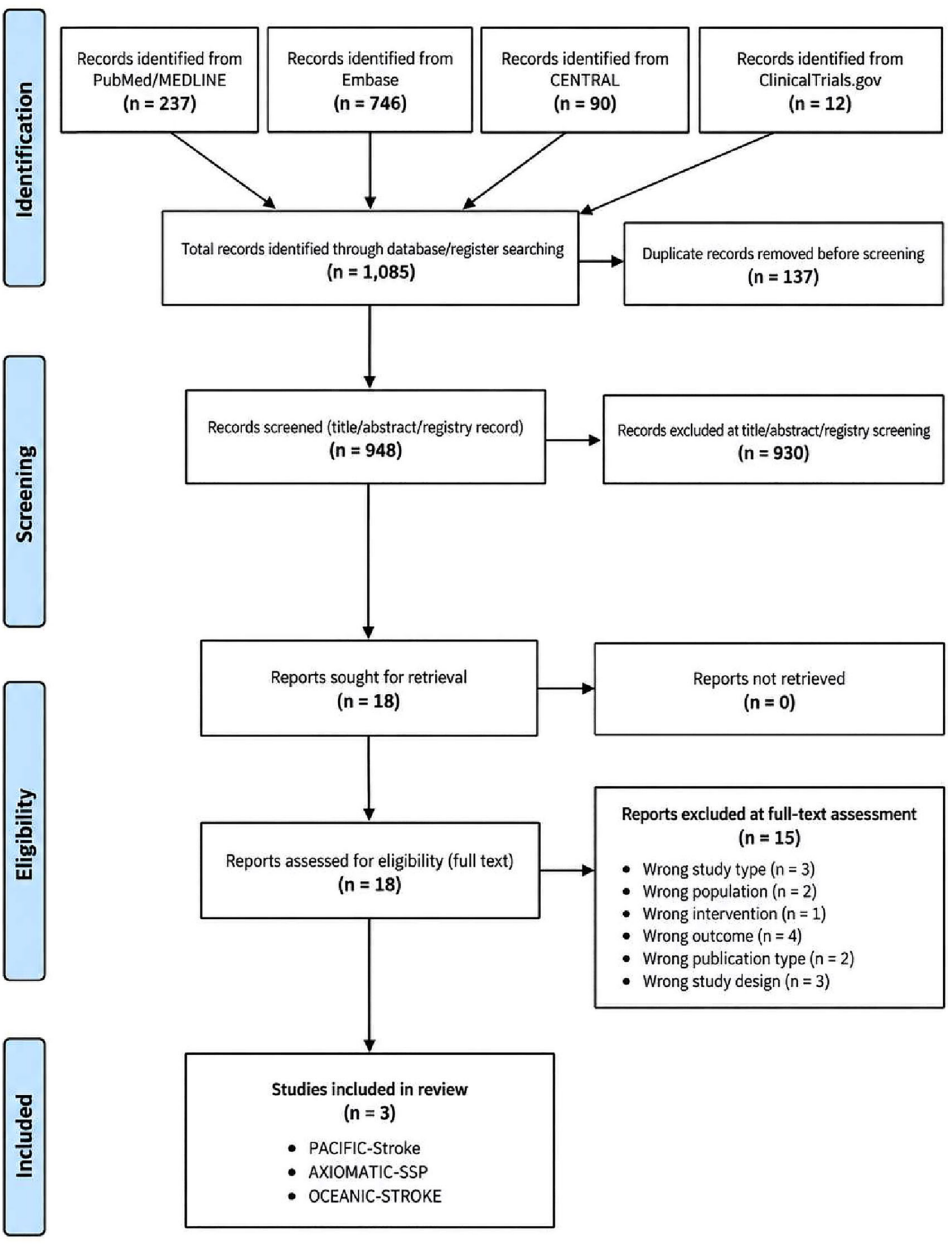
PRISMA flow diagram of study selection.

### 2.5 Data Extraction

Data were independently extracted by two reviewers (HN and AA) using a standardized data‐extraction form. Variables extracted showed trial name, year of publication, study phase, setting, country and center of involvement, sample size, inclusion/exclusion criteria, triggering event, intervention drug and dose, comparator, baseline antiplatelet therapy, duration of treatment, follow‐up duration, definitions of the outcome, counts of events, and denominators used in the analyses (Table 1, Table S2).

**TABLE 1:** Key study characteristics of included randomized controlled trials. Background antiplatelet therapy was protocol‐specified in each trial and could include single or dual antiplatelet therapy according to trial design and investigator discretion.

Study (trial and phase)	Countries	Total randomized, n	Population	Intervention arms evaluated in original trial	Intervention selected for primary meta‐analysis	Participants in primary efficacy comparison, n (FXIa inhibitor/placebo)	Comparator	Background antiplatelet therapy	Treatment/follow‐up duration
Sharma et al. (2026) (OCEANIC‐STROKE; Phase 3)	37	12,327	Adults ≥ 18 years with recent noncardioembolic ischemic stroke (NIHSS ≤ 15) or high‐risk TIA randomized within 72 h of symptom onset; participants were required to have a nonlacunar infarct, a history of atherosclerosis, or evidence of cerebrovascular atherosclerotic plaque	Asundexian 50 mg once daily	Asundexian 50 mg once daily	12,327 (6162/6165)	Matching placebo	Planned single or dual antiplatelet therapy according to the treating physician; randomization was stratified by planned antiplatelet regimen	Treatment continued until common trial completion; median follow‐up 567 days
Sharma et al. (2024) (AXIOMATIC‐SSP; Phase 2)	27	2366	Adults ≥ 40 years with recent ischemic stroke (NIHSS ≤ 7) or high‐risk TIA within 48 h, with qualifying imaging evidence of atherosclerotic plaque, ulceration, or thrombus	Milvexian 25 mg once daily; 25 mg twice daily; 50 mg twice daily; 100 mg twice daily; or 200 mg twice daily^a^	Milvexian 25 mg twice daily	1009 (318/691)	Matching placebo	Aspirin plus clopidogrel for the initial 21 days, followed by aspirin alone through day 90, according to trial protocol	90‐day double‐blind treatment period
Shoamanesh et al. (2022) (PACIFIC‐Stroke; Phase 2b)	23	1808	Adults ≥ 45 years with acute noncardioembolic ischemic stroke randomized within 48 h of symptom onset; minor or moderate stroke according to trial NIHSS criteria	Asundexian 10 mg, 20 mg, or 50 mg once daily	Asundexian 50 mg once daily	903 (447/456)	Matching placebo	Usual protocol‐directed antiplatelet therapy; randomization was stratified according to anticipated single versus dual antiplatelet therapy	26–52 weeks of treatment, with an additional follow‐up approximately 2 weeks after treatment discontinuation

*Note*: Key characteristics of the included randomized, double‐blind, placebo‐controlled trials. Across all treatment arms, the three trials randomized 16,501 participants. The primary selected‐dose efficacy meta‐analysis included 14,239 participants, comprising 6927 participants receiving an oral factor XIa inhibitor and 7312 receiving placebo. For the two multiarm phase 2 dose‐ranging trials, a single active‐treatment regimen was included in the primary analysis to avoid repeated inclusion of the shared placebo group: asundexian 50 mg once daily from PACIFIC‐Stroke and milvexian 25 mg twice daily from AXIOMATIC‐SSP. The corresponding selected‐dose populations were 447 versus 456 participants in PACIFIC‐Stroke and 318 versus 691 participants in AXIOMATIC‐SSP; OCEANIC‐STROKE randomized 6162 versus 6165 participants to asundexian 50 mg once daily and placebo, respectively.

Abbreviations: BID, twice daily; FXIa, activated factor XI; NIHSS, National Institutes of Health Stroke Scale; QD, once daily; TIA, transient ischemic attack.

^a^ AXIOMATIC‐SSP also contained small terminated once‐daily milvexian dose groups during the dose‐ranging program; these were not included in the primary selected‐dose comparison. The phase‐2 protocol specified a 90‐day double‐blind treatment period and protocol‐directed aspirin plus clopidogrel initially, followed by aspirin alone.

Data were extracted from the main reports, protocols, and supplementary appendices. Discrepancies were resolved in consensus with third reviewer (MAA) by re‐examination of the source reports and consensus between reviewers. For efficacy outcomes, randomized or intention‐to‐treat populations were used whenever available. For safety outcomes, analyses used the trial‐defined safety population, generally comprising participants who received at least one dose of study medication (Shoamanesh et al. 2022; Sharma et al. 2024, 2026). Consequently, denominators differed between efficacy and safety analyses where randomized participants did not receive study treatment.

### 2.6 Outcomes

The main efficacy endpoint was recurrent symptomatic ischemic stroke, defined and adjudicated by each trial. Symptomatic recurrent ischemic stroke was contributed by PACIFIC‐Stroke at the prespecified 26‐week assessment, new ischemic stroke was contributed during the 90‐day treatment period by AXIOMATIC‐SSP, and adjudicated ischemic stroke was contributed during the full trial follow‐up by OCEANIC‐STROKE. Randomized denominators were used for all comparisons selected, including the full randomized. There was no attempt to replace PACIFIC‐Stroke's prespecified 26‐week component with its later end‐of‐study ischemic stroke count in the primary analysis.

The most important safety outcome was the occurrence of major bleeding as defined by the International Society on Thrombosis and Haemostasis (ISTH) criteria. Harmonization of safety outcomes was done based on the most relevant clinically similar category as bleeding definitions were not the same across the trials included. Harmonized ISTH major‐bleeding data were available for all three trials, including the supplementary ISTH analysis from AXIOMATIC‐SSP. The AXIOMATIC‐SSP Bleeding Academic Research Consortium (BARC) type 3 or 5 endpoint was not used in the primary pooled major‐bleeding analysis (Shoamanesh et al. 2022; Sharma et al. 2024, 2026). In secondary outcomes, the definitions used in the trials remained as they were set but only were mapped to the same meta‐analysis category if they were deemed clinically similar. Definitions, extracted endpoints, and decisions concerning harmonization for each trial are summarized in Table S3.

The secondary efficacy outcome was all‐cause mortality. Secondary safety outcomes included major or clinically relevant non‐major bleeding, clinically relevant non‐major bleeding alone, minor or non‐actionable bleeding, intracranial or intracerebral hemorrhage, and fatal bleeding. Outcomes were extracted according to the definitions reported in each trial. In AXIOMATIC‐SSP, the ISTH major‐or‐clinically relevant‐non‐major bleeding composite included bleeding associated with on‐treatment adverse events or asymptomatic intracerebral hemorrhage detected on the day‐90 MRI assessment.

Minor or non‐actionable bleeding was defined by PACIFIC‐Stroke and OCEANIC‐STROKE and the closest comparable category was BARC type 1 non‐actionable bleeding for AXIOMATIC‐SSP. Definitions of intracranial bleeding also varied between trials—primary intracerebral hemorrhage in PACIFIC‐Stroke, modified BARC type 3c intracranial hemorrhage in AXIOMATIC‐SSP, and symptomatic intracranial hemorrhage, which included intracerebral hemorrhage in OCEANIC‐STROKE. In the interpretation of pooled estimates, and where applicable, in the assessment of the certainty of evidence, these differences in definitions were taken into account (Shoamanesh et al. 2022; Sharma et al. 2024, 2026).

In addition, functional outcomes—as measured by the modified Rankin Scale (mRS)—were sought but suitable arm‐level mRS data were not available for quantitative pooling across all trials included.

### 2.7 Risk‐of‐Bias Assessment

Two reviewers (MA, HN) independently assessed risk of bias using the Cochrane Risk of Bias 2 tool (Sterne et al. 2019). The domains assessed were those related to bias caused by the randomization, deviations from intended interventions, missing outcome data, measurement of outcomes, and selection of the outcome reported. Each included trial and outcome received an overall judgement of low risk, some concerns, or high risk. Differences were settled by consensus or by calling in a third referee (MAA). The assessment was shown graphically on the traffic light and summary plots, created with the Robvis tool (McGuinness and Higgins 2021) (Figure 2).

**FIGURE 2:**
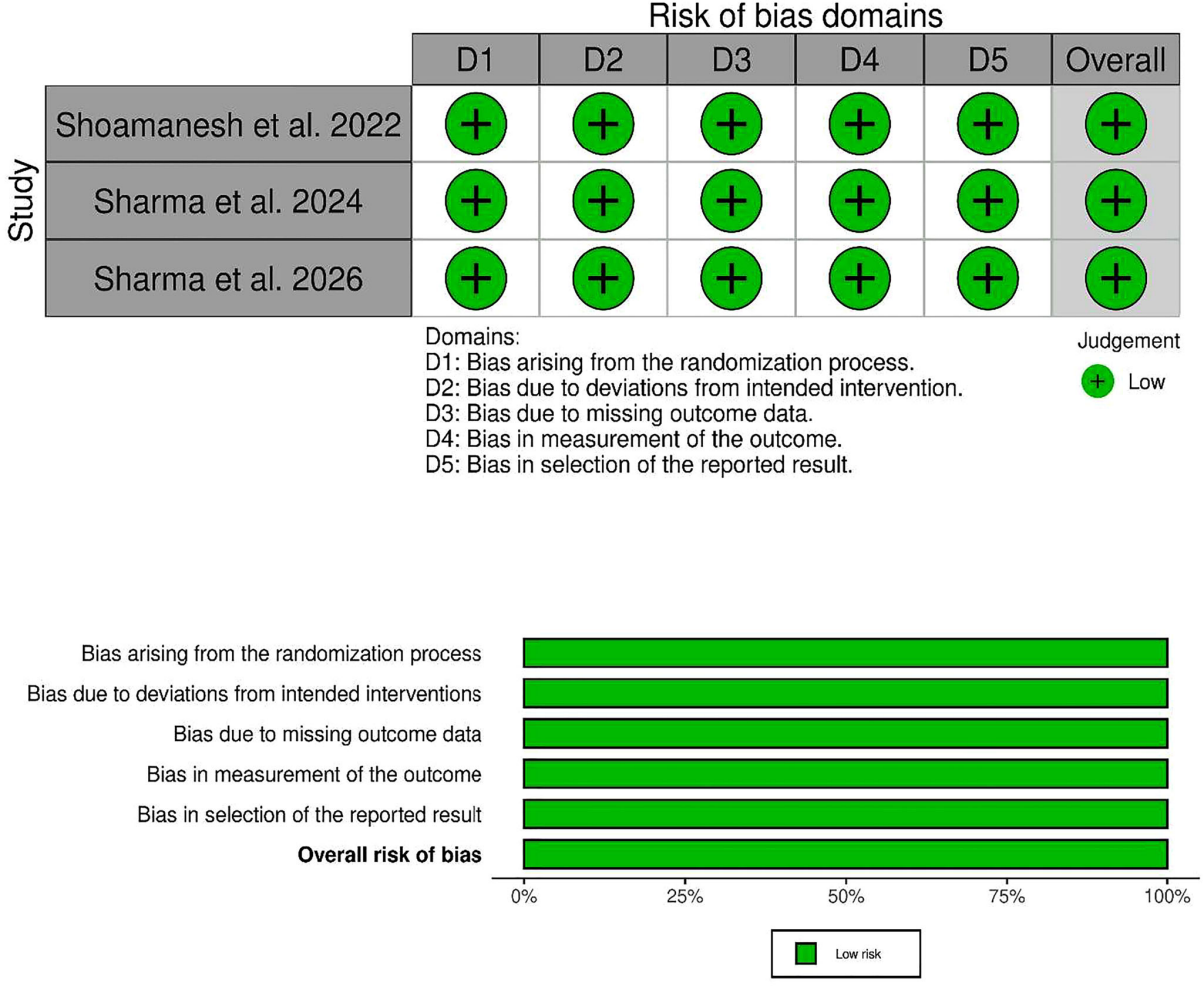
Risk‐of‐bias assessment of included randomized controlled trials by (a) traffic‐light plot and (b) summary plot.

### 2.8 GRADE Assessment

The certainty of evidence for each patient‐important outcome was evaluated using the Grading of Recommendations Assessment, Development and Evaluation (GRADE) approach (Guyatt et al. 2008). Initial evidence was rated as high certainty, and assessed in five domains: risk of bias, inconsistency, indirectness, imprecision, and publication bias. The level of certainty was classified as high, moderate, low or very low. The tables of summary of findings were created with GRADEpro GDT.2.9 (Evidence Prime 2026).

### 2.9 Statistical Analysis

Statistical analyses and forest plots were generated using Review Manager (RevMan), version 5.4.1(The Cochrane Collaboration 2020). For dichotomous outcomes, treatment effects were expressed as risk ratios (RRs) with 95% confidence intervals (CIs). Study‐specific log risk ratios for recurrent ischemic stroke, all‐cause mortality, major bleeding, major or clinically relevant non‐major bleeding, clinically relevant non‐major bleeding, and minor or non‐actionable bleeding were pooled using an inverse‐variance random‐effects method. Because intracranial or intracerebral hemorrhage and fatal bleeding were rare and included zero‐event treatment arms, these outcomes were analyzed using the Mantel–Haenszel random‐effects method. Pooled treatment effects were reported with conventional Wald‐type 95% confidence intervals based on the normal approximation. Hartung–Knapp adjustments were not applied. The use of Mantel–Haenszel methods for sparse outcomes was intended to reduce reliance on the large‐sample variance approximations required by conventional inverse‐variance methods (Deeks et al. 2019). An RR below 1.0 favored oral FXIa inhibition for recurrent ischemic stroke, whereas an RR above 1.0 indicated a higher risk of the assessed adverse or safety outcome with FXIa inhibition.

For the all‐dose sensitivity analysis, event counts and denominators from all eligible maintained active‐dose groups within each multiarm phase 2 trial were summed to create a single aggregated active‐treatment group. The corresponding shared placebo group was included only once, thereby avoiding duplicate use of control participants. A single trial‐level risk ratio was then calculated for each aggregated active‐treatment‐versus‐placebo comparison and pooled using the same random‐effects framework as the primary analysis.

Statistical heterogeneity was evaluated using Cochran's χ^2^ test, the between‐study variance (τ^2^), and the I^2^ statistic. I^2^ values of approximately < 25%, 25%–50%, and > 50% were interpreted as indicating low, moderate, and substantial heterogeneity, respectively (Deeks et al. 2019). Because the heterogeneity test has limited power when few studies are included, a *p* value < 0.10 was considered indicative of statistically significant heterogeneity. For the overall treatment effect, two‐sided *p* values < 0.05 were considered statistically significant (Page et al. 2019).

For outcomes with zero events in one treatment arm, RevMan's standard continuity correction was applied. Trials with zero events in both treatment groups were included in descriptive event totals but did not contribute to the pooled RR. Accordingly, AXIOMATIC‐SSP did not contribute statistical weight to the fatal‐bleeding estimate because no fatal bleeding events occurred in either selected treatment group. Formal assessment of publication bias using funnel plots or statistical tests was not performed because fewer than 10 studies were available for each outcome (Page et al. 2019).

Subgroup analyses were performed according to the FXIa inhibitor evaluated, categorized as asundexian or milvexian. Subgroup differences were assessed using the interaction test in RevMan. Because the milvexian subgroup was represented by a single phase 2 trial with relatively few events, subgroup findings were considered exploratory. We also sought subgroup data according to background single versus dual antiplatelet therapy. A quantitative subgroup meta‐analysis was planned only where trial‐level treatment‐effect estimates or arm‐specific event counts were available separately for both antiplatelet strata.

Trial sequential analysis (TSA) was additionally performed for the primary efficacy outcome of recurrent ischemic stroke and the primary safety outcome of ISTH major bleeding using Trial Sequential Analysis software (version 0.9.5.10 Beta). O'Brien–Fleming alpha‐spending trial‐sequential monitoring boundaries were applied using a two‐sided type I error of 5% and 90% power. Diversity‐adjusted required information sizes were estimated using a random‐effects model with model‐variance‐based heterogeneity correction. For recurrent ischemic stroke, an anticipated relative risk reduction of 20% was selected as a clinically meaningful moderate treatment effect rather than being derived from the observed pooled estimate; this assumption was also more conservative than the 26% relative reduction observed in the primary meta‐analysis. The control‐event proportion was 7.92%. For ISTH major bleeding, an anticipated 20% relative increase was selected as a clinically meaningful moderate harm threshold for an adjunctive antithrombotic therapy rather than being derived from the observed pooled estimate, with an anticipated intervention‐event proportion of 1.90% and a control‐event proportion of 1.58%. Futility was assessed using O'Brien–Fleming beta‐spending inner‐wedge boundaries. Trials were entered chronologically according to year of publication.

## 3 Results

### 3.1 Study Selection

A total of 1085 records were identified through database and trial‐registry search: PubMed/MEDLINE (n = 237), Embase (n = 746), Cochrane Library/CENTRAL (n = 90), and ClinicalTrials.gov (n = 12). A total of 137 duplicate records were removed and 948 records were then screened by their title, abstract, or registry‐record, with 930 being excluded. Eighteen reports were requested for retrieval, and all were retrieved and evaluated for eligibility. There were 15 reports excluded due to wrong study type (n = 3), wrong population (n = 2), wrong intervention (n = 1), wrong outcome (n = 4), wrong publication type (n = 2), and wrong study design (n = 3). Three RCTs were found that met the criteria for inclusion: PACIFIC‐Stroke, AXIOMATIC‐SSP, and OCEANIC‐STROKE. The complete study‐selection process is shown in Figure 1.

### 3.2 Study and Participant Characteristics

The three included trials randomized 16,501 participants across all active‐dose and placebo groups. Of these, 14,239 participants contributed to the primary selected‐dose efficacy meta‐analysis, including 6927 assigned to an oral FXIa inhibitor and 7312 assigned to placebo. The difference between total trial enrollment and the primary‐analysis population reflects the use of one selected active‐dose arm from each multiarm phase 2 trial, while the full randomized comparison from OCEANIC‐STROKE was retained.

PACIFIC‐Stroke contributed 447 participants receiving asundexian 50 mg once daily and 456 receiving placebo; AXIOMATIC‐SSP contributed 318 participants receiving milvexian 25 mg twice daily and 691 receiving placebo; and OCEANIC‐STROKE contributed 6162 participants receiving asundexian 50 mg once daily and 6165 receiving placebo. OCEANIC‐STROKE was a phase 3 trial conducted across 37 countries, whereas PACIFIC‐Stroke and AXIOMATIC‐SSP were phase 2 dose‐ranging trials evaluating asundexian and milvexian, respectively. Background antiplatelet therapy was continued according to each trial protocol. Follow‐up ranged from 90 days in AXIOMATIC‐SSP to a median of 567 days in OCEANIC‐STROKE. Key study characteristics, including the distinction between overall trial enrollment and the population contributing to the primary analysis, are summarized in Table 1.

Across the selected treatment comparisons, mean or median age ranged from approximately 67 to 72 years, and roughly two‐thirds of participants were male. Hypertension was the most commonly reported comorbidity. PACIFIC‐Stroke enrolled only participants with a qualifying ischemic stroke, whereas high‐risk TIA accounted for approximately 24% of participants in AXIOMATIC‐SSP and 5% in OCEANIC‐STROKE. Detailed baseline characteristics are presented in Table S2.

### 3.3 Functional Outcomes

There was some variation in how the trials reported functional outcome. The definition of disabling stroke by PACIFIC‐Stroke was ≥ 4 mRS, and by OCEANIC‐STROKE was ≥ 3 mRS at 90 days after the recurrent stroke or a one‐point rise in those who had a pre‐stroke mRS ≥ 3. For functional‐outcome data, AXIOMATIC‐SSP collected mRS longitudinally, while similar arm‐level functional‐outcome data were not available. No quantitative meta‐analysis of functional outcome (measured using mRS) was thus carried out.

### 3.4 Risk of Bias

According to the current RoB 2 assessment, all three trials were judged to have a low overall risk of bias. The trials used centralized randomization, double‐blinding, matching placebo controls, and blinded adjudication of major clinical outcomes. Domain‐level RoB 2 judgments are presented in Figure 2.

### 3.5 Primary Efficacy Outcome: Recurrent Ischemic Stroke

There were three trials reporting recurrent ischemic stroke including 14,239 randomized patients. 413 of 6927 oral FXIa inhibitor recipients had events, compared to 579 of 7312 who received placebo. Oral FXIa inhibition significantly lowered the risk of recurrent ischemic stroke, when compared with placebo (RR 0.74, 95% CI 0.65–0.84; I^2^ = 0%; *p *< 0.00001).

In the exploratory drug‐specific subgroup analysis, the pooled effect estimate was in favor of asundexian (RR 0.74, 95% CI 0.66–0.84; I^2^ = 0%) and for milvexian, the effect estimate was directionally similar but was imprecise and not statistically significant (RR 0.69, 95% CI 0.36–1.30). There was no evidence of a difference between the asundexian and milvexian subgroups (*p* for subgroup difference = 0.81). Milvexian was represented by a single trial in the milvexian subgroup, however, and the analysis was heavily impacted by OCEANIC‐STROKE (Figure 3a).

FIGURE 3Forest plot comparing oral factor XIa inhibitors with placebo for the following outcomes with exploratory subgroups according to asundexian and milvexian: (a) Recurrent ischemic stroke. (b) All‐cause mortality. (c) Major bleeding. (d) Major or clinically relevant non‐major bleeding. (e) Clinically relevant non‐major bleeding. (f) Minor bleeding. (g) Intracranial or intracerebral hemorrhage. (h) Fatal bleeding. Risk ratios were pooled using inverse‐variance random‐effects models for recurrent ischemic stroke, all‐cause mortality, major bleeding, major or clinically relevant non‐major bleeding, clinically relevant non‐major bleeding, and minor bleeding, whereas intracranial or intracerebral hemorrhage and fatal bleeding were pooled using Mantel–Haenszel random‐effects models. Values below 1.0 favor factor XIa inhibition. Efficacy outcomes are based on randomized/intention‐to‐treat populations; safety outcomes are based on trial‐defined treated safety populations. Accordingly, denominators may differ across outcomes.

**Figure:**
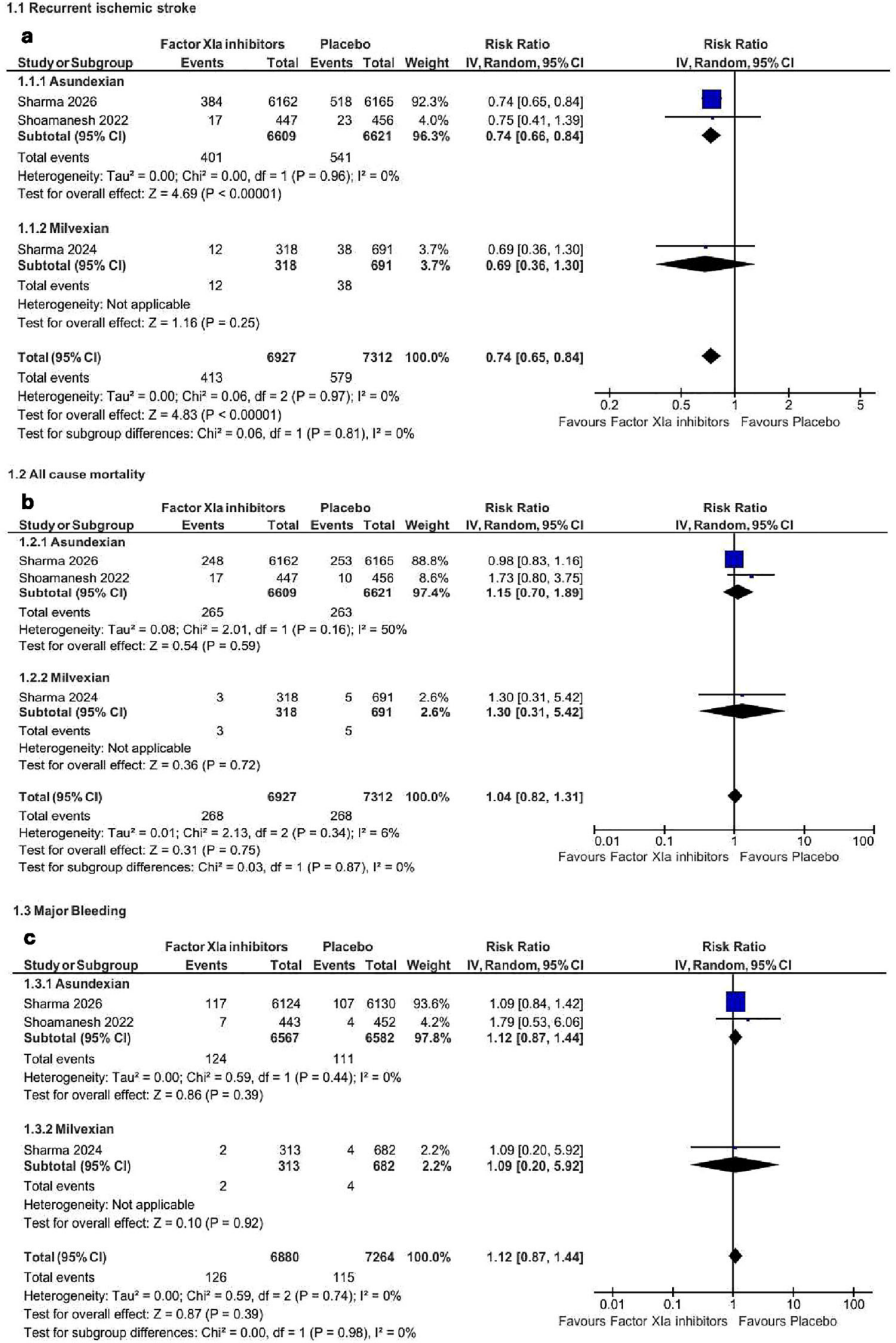

**Figure:**
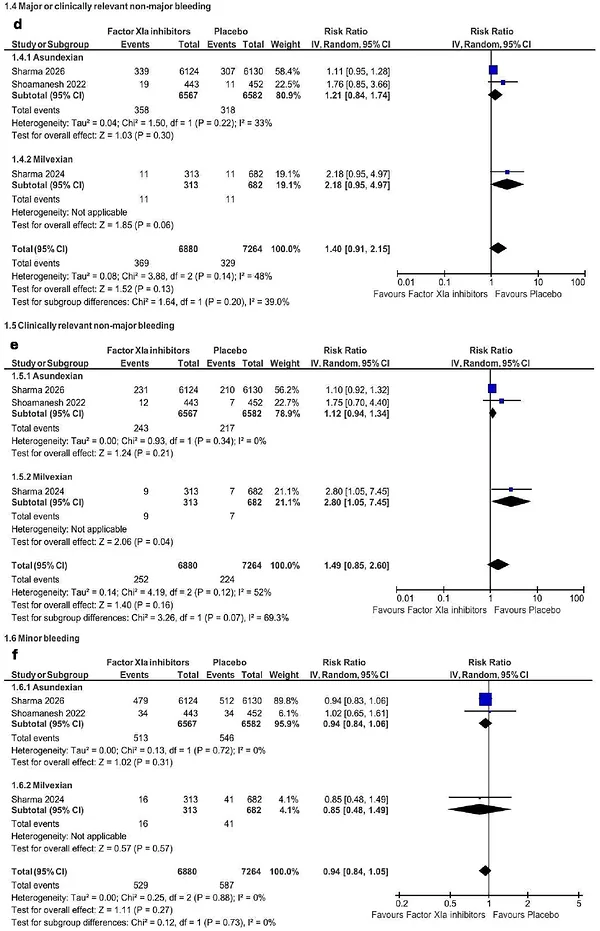

**Figure:**
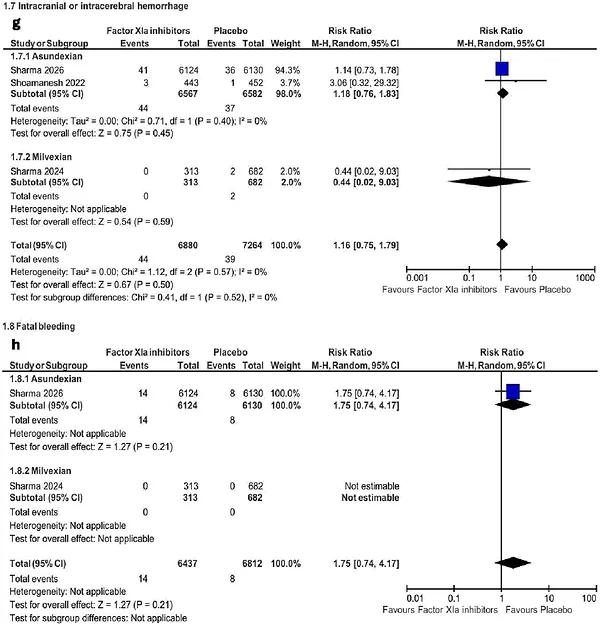

### 3.6 Secondary Efficacy Outcome: All‐Cause Mortality

In all three trials all‐cause mortality was reported. Overall, there were 268 deaths in 6927 patients treated with an FXIa inhibitor and 268 deaths in 7312 patients taking placebo. There was no statistically significant difference between groups (RR 1.04, 95% CI 0.82–1.31; I^2^ = 6%; *p* = 0.75).

In the subgroup analysis, mortality did not differ significantly with asundexian (RR 1.15, 95% CI 0.70–1.89; I^2^ = 50%) or milvexian (RR 1.30, 95% CI 0.31–5.42). The mortality effect was not different across the various FXIa inhibitors (*p* for subgroup difference = 0.87) (Figure 3b).

### 3.7 Primary Safety Outcome: Major Bleeding

In all three trials, there was a report of major bleeding in 14,144 people in the safety populations. In 126 of 6880, who also got an FXIa inhibitor, events happened, compared with 115 of 7264 who got the placebo. There was no statistical difference between groups in major bleeding (RR 1.12, 95% CI 0.87–1.44), but the confidence interval contained the possibility of a clinically important rise in bleeding.

The estimates were similar for asundexian (RR 1.12, 95% CI 0.87–1.44; I^2^ = 0%) and milvexian (RR 1.09, 95% CI 0.20–5.92), with no evidence of a subgroup difference (*p* for subgroup difference = 0.98). This is due to the limited number of major‐bleeding events recorded in AXIOMATIC‐SSP (Figure 3c) which makes the milvexian estimate very imprecise.

### 3.8 Secondary Safety Outcomes

#### 3.8.1 Major or Clinically Relevant Non‐Major Bleeding

The rate of major or clinically relevant non‐major bleeding was 369/6880 (5.3%) in those who received an FXIa inhibitor and 329/7264 (4.5%) in those who received placebo. No statistical difference for major or clinically relevant non‐major bleeding was found (RR 1.40, 95% CI 0.91–2.15); the confidence interval was consistent with a clinically important increase in bleeding.

The pooled estimate was RR 1.21 (95% CI 0.84–1.74; I^2^ = 33%) for asundexian and RR 2.18 (95% CI 0.95–4.97) for milvexian. No evidence of a difference in treatment effect between the two agents was found through the test for subgroup differences (*p* = 0.20) (Figure 3d).

#### 3.8.2 Clinically Relevant Non‐Major Bleeding

There were 252 clinically relevant non‐major bleeding events among 6880 participants receiving FXIa inhibitors and 224 among 7264 participants receiving placebo. The difference was not statistically significant (RR 1.49, 95% CI 0.85–2.60), although the confidence interval remained compatible with a clinically important increase in bleeding risk.

The asundexian subgroup estimate was RR 1.12 (95% CI 0.94–1.34; I^2^ = 0%). In AXIOMATIC‐SSP, the milvexian estimate was RR 2.80 (95% CI 1.05–7.45), based on 16 clinically relevant non‐major bleeding events. The test for subgroup interaction was not statistically significant (*p* for subgroup difference = 0.07). The milvexian subgroup was represented by a single trial, limiting the precision and interpretability of the subgroup comparison (Figure 3e).

#### 3.8.3 Minor or Non‐Actionable Bleeding

Minor or non‐actionable bleeding occurred in 529 of 6880 participants receiving an FXIa inhibitor and in 587 of 7264 receiving placebo. There was no statistically significant difference between groups (RR 0.94, 95% CI 0.84–1.05; I^2^ = 0%; *p* = 0.27).

No significant effect was observed with asundexian (RR 0.94, 95% CI 0.84–1.06; I^2^ = 0%) or milvexian (RR 0.85, 95% CI 0.48–1.49), and there was no evidence of a subgroup difference (*p* = 0.73). AXIOMATIC‐SSP reported BARC type 1 non‐actionable bleeding as the closest corresponding category to minor bleeding; therefore, this pooled outcome should be interpreted with recognition that definitions were not identical across trials (Figure 3f).

#### 3.8.4 Intracranial or Intracerebral Hemorrhage

Intracranial or intracerebral hemorrhage occurred in 44 of 6880 participants receiving an FXIa inhibitor and 39 of 7264 receiving placebo. No statistically significant difference was observed between groups (RR 1.16, 95% CI 0.75–1.79; I^2^ = 0%; *p* = 0.50). The asundexian subgroup estimate was RR 1.18 (95% CI 0.76–1.83; I^2^ = 0%), whereas the estimate from the single milvexian trial was RR 0.44 (95% CI 0.02–9.03). No significant subgroup difference was detected (*p* = 0.52).

Definitions of intracranial bleeding differed across the included trials. PACIFIC‐Stroke reported primary intracerebral hemorrhage, AXIOMATIC‐SSP reported modified BARC type 3c intracranial hemorrhage including symptomatic hemorrhagic transformation, and OCEANIC‐STROKE reported symptomatic intracranial hemorrhage. These differences, together with the small number of events and wide confidence intervals, limited the precision and direct comparability of the pooled estimate (Table S3; Figure 3g).

#### 3.8.5 Fatal Bleeding

Fatal bleeding was separately reported in AXIOMATIC‐SSP and OCEANIC‐STROKE. No fatal bleeding occurred in either selected AXIOMATIC‐SSP group, leaving OCEANIC‐STROKE as the only trial with an estimable effect. Fatal bleeding occurred in 14 of 6124 participants receiving asundexian and in 8 of 6130 receiving placebo. Fatal bleeding was rare, and the pooled estimate was highly imprecise (RR 1.75, 95% CI 0.74–4.17). The available data were therefore insufficient to exclude either a reduction or a clinically important increase in fatal bleeding.

A subgroup‐difference test could not be performed because the milvexian study had zero events in both groups and contributed no statistical weight. Between‐study heterogeneity was therefore not estimable (Figure 3h).

### 3.9 Subgroup Analyses by FXIa Inhibitor

Across the evaluated efficacy and safety outcomes, no statistically significant subgroup interaction was observed between asundexian and milvexian. For clinically relevant non‐major bleeding, the interaction *p* value was 0.07. The milvexian subgroup was represented by a single phase 2 trial. The corresponding inhibitor‐specific subgroup analyses for recurrent ischemic stroke, all‐cause mortality, major bleeding, major or clinically relevant non‐major bleeding, clinically relevant non‐major bleeding, minor bleeding, intracranial or intracerebral hemorrhage, and fatal bleeding are presented in Figure 3. Subgroup data according to background single versus dual antiplatelet therapy were not reported in a sufficiently consistent and extractable form across the included trials to permit quantitative pooling. OCEANIC‐STROKE stratified randomization according to planned single versus dual antiplatelet therapy, but comparable arm‐level subgroup data were not available across the other included trials. Therefore, no meta‐analysis according to background antiplatelet regimen was performed.

### 3.10 Certainty of Evidence by GRADE Assessment

The certainty of evidence was judged to be high for recurrent ischemic stroke, moderate for all‐cause mortality, major bleeding, major or clinically relevant non‐major bleeding, clinically relevant non‐major bleeding, minor bleeding, and low for intracranial or intracerebral hemorrhage and fatal bleeding using the GRADE framework. Imprecision and variation in outcome definitions in the case of intracranial bleeding were the main issues associated with downgrading. Specific GRADE judgments and explanations are given in Table 2.

**TABLE 2:** Summary of findings and GRADE certainty assessment.

Outcome	Risk with placebo	Risk with oral factor XIa inhibitors	Relative effect	Participants	Certainty of evidence	Interpretation
Recurrent ischemic stroke	79 per 1000	59 per 1000	RR 0.74 (0.65–0.84)	14,239; 3 RCTs	High^a^, ^b^, ^c^, ^d^	The pooled randomized evidence indicates a reduction in recurrent ischemic stroke with oral FXIa inhibition added to background antiplatelet therapy; however, the estimate is predominantly driven by OCEANIC‐STROKE, and certainty regarding generalization across individual FXIa inhibitors is more limited.
All‐cause mortality	37 per 1000	38 per 1000	RR 1.04 (0.82–1.31)	14,239; 3 RCTs	Moderate^e^, ^f^, ^g^, ^h^	Oral factor XIa inhibitors probably result in little to no difference in all‐cause mortality, although clinically important benefit or harm cannot be excluded.
Major bleeding	16 per 1000	18 per 1000	RR 1.12 (0.87–1.44)	14,144; 3 RCTs	Moderate^i^, ^j^, ^k^, ^l^	No statistically significant difference was detected, but the confidence interval remained compatible with a clinically important increase in major bleeding; safety equivalence was therefore not established.
Major or clinically relevant non‐major bleeding	45 per 1000	63 per 1000	RR 1.40 (0.91–2.15)	14,144; 3 RCTs	Moderate^m^, ^n^, ^o^, ^p^	No statistically significant difference was detected, but a clinically important increase in bleeding could not be excluded.
Clinically relevant non‐major bleeding	31 per 1000	46 per 1000	RR 1.49 (0.85–2.60)	14,144; 3 RCTs	Moderate^q^, ^r^, ^s^, ^t^	Oral factor XIa inhibitors probably result in little to no difference in clinically relevant non‐major bleeding, although a clinically important increase cannot be excluded. The estimate was imprecise and compatible with a substantial increase in clinically relevant non‐major bleeding.
Intracranial or intracerebral hemorrhage	5 per 1000	6 per 1000	RR 1.16 (0.75–1.79)	14,144; 3 RCTs	Low^u^, ^v^, ^w^, ^x^	Oral factor XIa inhibitors may result in little to no difference in intracranial or intracerebral hemorrhage, but the evidence is uncertain. The effect remains uncertain because of rare events, wide confidence intervals, and variation in endpoint definitions.
Fatal bleeding	1 per 1000	2 per 1000	RR 1.75 (0.74–4.17)	13,249; 2 RCTs	Low^y^, ^z^, ^aa^, ^ab^	The effect of oral factor XIa inhibitors on fatal bleeding is uncertain because the estimate is based on very few events. The effect remains highly uncertain because very few events occurred and the confidence interval was wide.
Minor or non‐actionable Bleeding	81 per 1000	76 per 1000	RR 0.94 (0.84–1.05)	14,144; 3 RCTs	Moderate^ac^, ^ad^, ^ae^, ^af^	Oral factor XIa inhibitors probably result in little to no difference in minor or non‐actionable bleeding.

^a^ All three trials were randomized, double‐blind, placebo‐controlled studies with blinded outcome adjudication. No important risk of bias was identified for recurrent ischemic stroke. Although statistical inconsistency was not observed, the pooled estimate was predominantly driven by the phase 3 OCEANIC‐STROKE trial of asundexian; therefore, the high‐certainty rating applies to the pooled treatment estimate in the studied population and should not be interpreted as evidence of identical efficacy across all oral FXIa inhibitors.

^b^ Study‐specific estimates were directionally consistent, statistical heterogeneity was absent (I^2^ = 0%; heterogeneity *p* = 0.97), and no important unexplained inconsistency was identified.

^c^ The included trials directly evaluated oral FXIa inhibitors plus background antiplatelet therapy versus matching placebo plus antiplatelet therapy in adults with recent noncardioembolic ischemic stroke or high‐risk TIA and reported the patient‐important outcome of recurrent ischemic stroke.

^d^ The analysis included 992 recurrent ischemic‐stroke events among 14,239 participants. The confidence interval excluded no effect and remained entirely consistent with a clinically important reduction in recurrence (RR 0.74, 95% CI 0.65–0.84).

^e^ All three studies were randomized, double‐blind, placebo‐controlled trials. Mortality is an objective outcome, and no important risk‐of‐bias concerns were identified.

^f^ Statistical heterogeneity was low (I^2^ = 6%; heterogeneity *p* = 0.34). Although the smaller phase 2 trials had point estimates above 1.0, their confidence intervals were wide and overlapped with the OCEANIC‐STROKE estimate.

^g^ The trials directly evaluated the intervention, comparator, and target population specified in the review question, and all‐cause mortality is a directly patient‐important outcome.

^h^ The pooled confidence interval crossed the line of no effect and included both a potentially important mortality reduction and a potentially important increase (RR 1.04, 95% CI 0.82–1.31). Thus, clinically meaningful benefit or harm could not be excluded.

^i^ All three trials were randomized, double‐blind, placebo‐controlled studies with adjudicated bleeding outcomes. No important risk‐of‐bias concerns were identified for major bleeding.

^j^ No important statistical heterogeneity was observed (I^2^ = 0%; heterogeneity *p* = 0.74), and the study‐specific estimates were broadly compatible.

^k^ The trials directly evaluated oral FXIa inhibitors plus background antiplatelet therapy versus placebo plus background antiplatelet therapy in the target population. Harmonized ISTH major‐bleeding data were used across all three trials.

^l^ The confidence interval crossed the line of no effect and was compatible with both a modest reduction and a clinically important increase in major bleeding (RR 1.12, 95% CI 0.87–1.44). Therefore, meaningful harm could not be excluded.

^m^ All three studies were randomized, double‐blind, placebo‐controlled trials with adjudicated bleeding outcomes. No important risk‐of‐bias concerns were identified.

^n^ Moderate statistical heterogeneity was observed (I^2^ = 48%), but all study‐specific estimates were in the same direction, confidence intervals overlapped, and the test for subgroup differences between asundexian and milvexian was not statistically significant.

^o^ The studies directly evaluated oral FXIa inhibitors plus background antiplatelet therapy versus placebo plus background antiplatelet therapy in the target population and reported the patient‐important composite of major or clinically relevant non‐major bleeding.

^p^ The confidence interval crossed the line of no effect and was compatible with both little or no difference and a clinically important increase in bleeding (RR 1.40, 95% CI 0.91–2.15).

^q^ All three studies were randomized, double‐blind, placebo‐controlled trials with prospectively defined bleeding outcomes. No important risk‐of‐bias concerns were identified for clinically relevant non‐major bleeding.

^r^ Moderate statistical heterogeneity was observed (I^2^ = 52%), but all study‐specific point estimates were in the same direction, confidence intervals overlapped, and the heterogeneity test was not statistically significant. The apparent difference between asundexian and milvexian was based on a single milvexian trial and the interaction test did not reach statistical significance.

^s^ The included trials directly evaluated oral FXIa inhibitors plus background antiplatelet therapy versus placebo plus background antiplatelet therapy in the target population and reported clinically relevant non‐major bleeding.

^t^ The confidence interval crossed the line of no effect and included both a possible reduction and a large clinically important increase in clinically relevant non‐major bleeding (RR 1.49, 95% CI 0.85–2.60). Important harm therefore could not be excluded.

^u^ All three studies were randomized, double‐blind, placebo‐controlled trials with prospectively defined and adjudicated hemorrhagic outcomes. No important risk‐of‐bias concerns were identified.

^v^ No statistical heterogeneity was detected (I^2^ = 0%), although the analysis had limited power because intracranial hemorrhage was rare and only three trials were included.

^w^ Definitions varied across trials: PACIFIC‐Stroke reported primary intracerebral hemorrhage, AXIOMATIC‐SSP reported modified BARC type 3c intracranial hemorrhage including symptomatic hemorrhagic transformation, and OCEANIC‐STROKE reported symptomatic intracranial hemorrhage. These differences introduce important indirectness in the pooled outcome.

^x^ Only 83 total events occurred, and the confidence interval crossed the line of no effect and included both a potentially important reduction and increase in intracranial hemorrhage (RR 1.16, 95% CI 0.75–1.79).

^y^ The contributing trial was randomized, double‐blind, and placebo‐controlled, and fatal bleeding is an objective outcome. No important risk‐of‐bias concerns were identified.

^z^ Statistical inconsistency could not be meaningfully assessed because only one trial contributed estimable comparative data. No demonstrated inconsistency was identified.

^aa^ The contributing evidence directly evaluated oral FXIa inhibition plus background antiplatelet therapy versus placebo plus antiplatelet therapy in the target population and reported the patient‐important outcome of fatal bleeding.

^ab^ Only 22 fatal bleeding events occurred, and the confidence interval was extremely wide, crossing no effect and including both a possible reduction and more than a fourfold increase in fatal bleeding (RR 1.75, 95% CI 0.74–4.17). The estimate is therefore highly uncertain.

^ac^ All three studies were randomized, double‐blind, placebo‐controlled trials, and no important risk‐of‐bias concerns were identified.

^ad^ Study estimates were consistent, with no statistical heterogeneity (I^2^ = 0%).

^ae^ A one‐level downgrade is reasonable because the endpoint was not defined identically across trials.

^af^ The confidence interval was relatively narrow and excluded large clinically important benefit or harm (RR 0.94, 95% CI 0.84–1.05).

### 3.11 Sensitivity Analysis

During the all‐dose sensitivity analysis, 528 recurrent ischemic strokes were observed in 9149 patients taking an oral FXIa inhibitor and 579 in 7312 patients taking placebo. The pooled estimate was RR 0.77 (95% CI 0.68–0.86; I^2^ = 0%; *p *< 0.00001), compared with RR 0.74 (95% CI 0.65–0.84) in the primary selected‐dose analysis. The statistical weight of OCEANIC‐STROKE was 84.1% (Figure S1).

### 3.12 Trial Sequential Analysis

For recurrent ischemic stroke, the TSA calculated that 11,149 participants were needed. This sample size was met and the cumulative Z‐curve crossed both the conventional significance boundary and the O'Brien‐Fleming trial‐sequential monitoring boundary (Figure S2).

Estimated information size of 70,175 participants was required for ISTH major bleeding. This leaves a safety population of 14,144, which is about 20% of the required information size. The cumulative Z‐curve failed to cross the usual significance boundary or the O'Brien–Fleming trial‐sequential monitoring boundary but also did not cross the futility region (Figure S3).

## 4 Discussion

The use of oral FXIa inhibition in addition to protocol‐specified background antiplatelet therapy was shown to be significantly associated with decreased recurrent ischemic stroke in this meta‐analysis and systematic review of three randomized, double‐blind, and placebo‐controlled trials involving 14,239 patients with recent noncardioembolic ischemic stroke or high‐risk TIA. In the primary selected‐dose analysis, the relative reduction was 26% for recurrent ischemic stroke; this result was similar in the sensitivity analysis which also included all of the maintained active‐doses from the phase 2 trials. Importantly, the concordant all‐dose sensitivity analysis offers reassurance that the pooled efficacy estimate was not primarily driven by the selection of the individual phase 2 treatment arms as it was not prespecified in the registered protocol. The pooled efficacy estimate was largely from the OCEANIC‐STROKE trial, however, and the phase 2 trials individually were unable to give evidence of benefit for all dose regimens. Thus, the high level of GRADE certainty for the pooled recurrent‐stroke estimate does not mean that the overall treatment effect is also highly certain for each individual FXIa inhibitor. There was no statistically significant increase in major bleeding and the confidence interval did not rule out clinically important harm. The findings from the TSA also distinguished between the strength of the efficacy and safety data, as the recurrent‐stroke analysis had sufficient information size and crossed the trial‐sequential monitoring boundary, while the major‐bleeding analysis had only about one‐fifth of the required information size and did not cross either a monitoring or a futility boundary. Taken together, the available evidence supports a reduction in recurrent ischemic stroke with oral FXIa inhibition, whereas the effect on major bleeding remains uncertain. The absence of a statistically significant increase in bleeding should not be interpreted as evidence of bleeding neutrality, particularly because the major‐bleeding TSA remained substantially underpowered relative to the required information size.

These findings extend the available randomized evidence on FXIa inhibition after noncardioembolic ischemic stroke or high‐risk TIA. PACIFIC‐Stroke and AXIOMATIC‐SSP were phase 2 dose‐ranging trials that did not demonstrate significant dose–response reductions in their primary composite cerebral ischemic outcomes, although symptomatic ischemic stroke estimates generally favored selected active‐dose groups (Shoamanesh et al. 2022; Sharma et al. 2024). OCEANIC‐STROKE subsequently showed that asundexian 50 mg once daily reduced clinical ischemic stroke compared with placebo without a statistically significant increase in ISTH major bleeding (Sharma et al. 2026). Earlier meta‐analyses either predated the phase 3 OCEANIC‐STROKE trial or combined heterogeneous populations, indications, agents, and comparators (Palaiodimou et al. 2023; Zahid et al. 2025). The present analysis differs by focusing specifically on patients with recent noncardioembolic ischemic stroke or high‐risk TIA and by comparing oral FXIa inhibition with matching placebo on a background of antiplatelet therapy.

The observed decrease in recurrent ischemic stroke is a biologically plausible effect. FXI is involved in thrombin amplification and propagation, and seems to have a less crucial role in hemostasis than thrombin or FXa. Lower FXI activity has been associated with lower thrombotic risk and a relatively mild spontaneous bleeding phenotype, which supports the notion that lower FXI activity (or inhibition) may separate antithrombotic efficacy from bleeding risks (Hsu et al. 2021; Salomon et al. 2008; Georgi et al. 2019). The FXIa inhibitors evaluated in these stroke populations were used as add‐on agents to antiplatelet therapy instead of as alternatives to the standard antithrombotic agent therapy. This is significant because different components of thrombosis are involved and are targeted by different drugs such as antiplatelet agents and FXIa inhibitors.

The results should not be extrapolated to cardioembolic stroke or atrial fibrillation. In OCEANIC‐AF, asundexian was less effective than apixaban for preventing stroke or systemic embolism in patients with atrial fibrillation, despite causing less bleeding (Piccini et al. 2025). Therefore, the present findings apply only to the studied population: patients with recent noncardioembolic ischemic stroke or high‐risk TIA receiving background antiplatelet therapy. Patients with atrial fibrillation or another established indication for therapeutic anticoagulation should receive guideline‐directed oral anticoagulation rather than FXIa inhibition on the basis of these data (Kleindorfer et al. 2021; Piccini et al. 2025).

Interpretation of safety findings must be done carefully. There were no statistically significant differences between the groups for major bleeding, major or clinically relevant non‐major bleeding, clinically relevant non‐major bleeding, minor or non‐actionable bleeding, intracranial or intracerebral hemorrhage, fatal bleeding, or all‐cause death. Statistical non‐significance, however, should not be taken as proof of equivalency. Few events, large confidence intervals, and differences in outcome criteria between trials hampered a number of safety estimates. Only one trial contributed an estimable treatment impact for fatal bleeding, and intracranial and fatal bleeding were notably imprecise. TSA, where just about 20% of the necessary information size for significant bleeding had accumulated and neither the trial‐sequential monitoring nor the futility barrier had been achieved, added to this uncertainty. Thus, the absence of statistically significant harm should not be interpreted as proof of equivalent safety or as excluding a clinically important increase in major bleeding.

Clinical heterogeneity should also be considered. PACIFIC‐Stroke enrolled only patients with ischemic stroke, whereas AXIOMATIC‐SSP and OCEANIC‐STROKE included both ischemic stroke and high‐risk TIA populations (Shoamanesh et al. 2022; Sharma et al. 2024, 2026). The trials differed in treatment duration, follow‐up duration, qualifying‐event criteria, FXIa inhibitor evaluated, and background protocol‐specified antiplatelet therapy. Outcome definitions also varied for minor bleeding and intracranial hemorrhage. These differences may have contributed to the moderate heterogeneity observed for clinically relevant non‐major bleeding. In addition, OCEANIC‐STROKE contributed most of the statistical weight for several outcomes, meaning that the pooled results should be viewed mainly as confirmation and contextualization of the phase 3 evidence rather than as three equally weighted independent replications.

The similarity between the all‐dose and selected‐dose sensitivity estimates indicates that the choice of individual phase 2 treatment arms did not significantly affect the pooled efficacy result. OCEANIC‐STROKE continued to be the primary driver of the pooled estimate, nevertheless. The lack of a statistically significant interaction should not be taken as proof of comparable drug‐specific effects, even though subgroup analyzes by FXIa inhibitor did not reveal statistically significant treatment‐effect differences between asundexian and milvexian. Because the Asundexian subgroup was dominated by OCEANIC‐STROKE, while the milvexian subgroup was represented by a single phase 2 experiment with comparatively few events, these analyses were exploratory and underpowered. It should be considered hypothesis‐generating rather than proof of a real difference between agents because the interaction for clinically significant non‐major bleeding approached but did not reach conventional statistical significance. Asundexian and milvexian have not been directly compared in a head‐to‐head trial.

This review has several strengths, including restriction to randomized, double‐blind, placebo‐controlled trials; a focused clinical population; use of randomized populations for efficacy outcomes and treated safety populations for bleeding outcomes; harmonization of ISTH major bleeding across trials; avoidance of repeated counting of shared placebo groups; and an all‐dose sensitivity analysis. Limitations include the small number of trials, statistical dominance of OCEANIC‐STROKE, use of aggregate study‐level data, variation in follow‐up duration and outcome definitions, limited ability to assess dose–response relationships, and imprecision for rare bleeding outcomes. Fewer than 10 studies were available for each outcome, precluding meaningful assessment of publication bias. Comparable functional‐outcome data were not available across all trials, preventing pooled analysis of mRS‐based disability outcomes. In addition, the available aggregate data did not permit a pooled assessment of whether efficacy or bleeding risk differed according to background single versus dual antiplatelet therapy. Further randomized and real‐world studies are needed to clarify long‐term safety, dose‐specific effects, comparative efficacy across individual FXIa inhibitors, and the patient subgroups most likely to derive net clinical benefit.

## 5 Conclusion

Among patients with recent noncardioembolic ischemic stroke or high‐risk TIA receiving background antiplatelet therapy, pooled randomized evidence showed a reduction in recurrent ischemic stroke with oral FXIa inhibition, with a similar effect observed in the all‐dose sensitivity analysis. However, the efficacy estimate was predominantly driven by the phase 3 OCEANIC‐STROKE trial, and evidence supporting generalization across individual FXIa inhibitors remains limited. No statistically significant increase in major bleeding or other bleeding outcomes was detected, but the corresponding confidence intervals, particularly for uncommon serious bleeding events, remained compatible with clinically important harm and do not establish safety equivalence. Further randomized evidence is needed to clarify long‐term safety, dose‐specific effects, and the consistency of treatment benefit across individual FXIa inhibitors and patient subgroups.

## Author Contributions

**Mirza Ammar Arshad**: conceptualization, data curation, formal analysis, project administration, software, validation, supervision, and writing – original draft. **Hina Nadeem**: formal analysis, data curation, visualization, writing – original draft, writing – review and editing. **Maham Afzal**: formal analysis, software, visualization, validation, writing – review and editing. **Abbas Ali**: data curation, investigation, validation, writing – review and editing. **Rasheeda Shehroz**: writing – original draft, writing – review and editing. **Affan Khalid**: methodology, writing – original draft. **Zaiya Waseem**: methodology, writing – original draft, writing – review and editing. **Ahsan Raza Raja**: writing – original draft. **Eman Naeem Cheema**: writing – original draft, writing – review and editing. **FNU Hizbullah**: literature screening, data extraction. **Gulleena Junaid**: literature screening, data extraction. **Asad Ali Ahmed Cheema**: conceptualization, supervision, project administration, validation, writing – review and editing.

## Funding

The authors have nothing to report.

## Ethics Statement

The authors have nothing to report.

## Consent

The authors have nothing to report.

## Conflicts of Interest

The authors declare no conflicts of interest.

## Supporting information




**Supplementary Figure S1**: Sensitivity analysis of recurrent ischemic stroke using all maintained active‐dose groups
**Supplementary Figure S2**: Trial sequential analysis for recurrent ischemic stroke
**Supplementary Figure S3**: Trial sequential analysis for ISTH major bleeding
**Supplementary Table S1A**. Complete electronic search strategies used for database and trial‐registry searches
**Supplementary Table S1B**. PRISMA‐S reporting details for electronic information sources
**Supplementary Table S2**: Baseline Characteristics of the included randomized clinical trials
**Supplementary Table S3**: Trial‐specific definitions and harmonization of safety outcomes.

## Data Availability

All data analyzed in this study were obtained from the published reports cited in the manuscript.
