# Supplementary material for: Oral Factor XIa Inhibitors for Secondary Prevention After Noncardioembolic Ischemic Stroke or High‐Risk Transient Ischemic Attack: Systematic Review and Meta‐Analysis
Source: Brain Behav. 2026 Oct 22;16(10):e71846. doi: 10.1002/brb3.71846 (PMC13691415; doi:10.1002/brb3.71846)
Supplement: Supplementary file 1 [file BRB3-16-e71846-s001.docx]

**SUPPLEMENTARY FIGURES LEGEND**

**Supplementary Figure S1:** Sensitivity analysis of recurrent ischemic stroke using all maintained active-dose groups.

**Supplementary Figure S2:** Trial sequential analysis for recurrent ischemic stroke

**Supplementary Figure S3:** Trial sequential analysis for ISTH major bleeding

**SUPPLEMENTARY TABLES LEGEND**

**Supplementary Table S1A.** Complete electronic search strategies used for database and trial-registry searches

**Supplementary Table S1B.** PRISMA-S reporting details for electronic information sources

**Supplementary Table S2:** Baseline Characteristics of the included randomized clinical trials

**Supplementary Table S3:** Trial-specific definitions and harmonization of safety outcomes

**Supplementary Figure S1.** Sensitivity analysis of recurrent ischemic stroke using all maintained active-dose groups. Risk ratios for recurrent ischemic stroke were pooled using an inverse-variance random-effects model.

**Supplementary Figure S2:** Trial sequential analysis for recurrent ischemic stroke

Trial sequential analysis of recurrent ischemic stroke comparing oral factor XIa inhibitors with placebo. The cumulative Z-curve was evaluated against conventional significance boundaries and O’Brien–Fleming trial-sequential monitoring boundaries using a two-sided type I error of 5%, 90% power, an anticipated relative risk reduction of 20%, a control event proportion of 7.92%, and model-variance-based diversity adjustment. The anticipated 20% relative risk reduction was selected as a clinically meaningful moderate treatment effect and was not derived from the observed pooled estimate. The estimated required information size was 11,149 participants. The cumulative Z-curve crossed the trial-sequential monitoring boundary, and the accrued sample size exceeded the required information size.

**Supplementary Figure S3:** Trial sequential analysis for ISTH major bleeding

Trial sequential analysis of International Society on Thrombosis and Haemostasis (ISTH)-defined major bleeding comparing oral factor XIa inhibitors with placebo. The cumulative Z-curve was evaluated against conventional significance boundaries and O’Brien–Fleming trial-sequential monitoring boundaries using a two-sided type I error of 5%, 90% power, an anticipated 20% relative increase in major bleeding, a control event proportion of 1.58%, and model-variance-based diversity adjustment. The anticipated 20% relative increase was selected as a clinically meaningful moderate harm threshold for an adjunctive antithrombotic therapy and was not derived from the observed pooled estimate. The estimated required information size was 70,175 participants. The cumulative Z-curve did not cross the conventional significance boundary, the trial-sequential monitoring boundary, or the futility boundary, and the accrued sample size of 14,144 participants remained substantially below the required information size.

| **Database** | **Search components** | **Search strategy** |
| --- | --- | --- |
| **PubMed** | **Population: Stroke or transient ischemic attack** | ("Stroke"[MeSH Terms] OR "Brain Ischemia"[MeSH Terms] OR "Cerebral Infarction"[MeSH Terms] OR "Ischemic Attack, Transient"[MeSH Terms] OR stroke[Title/Abstract] OR strokes[Title/Abstract] OR "ischemic stroke"[Title/Abstract] OR "ischemic strokes"[Title/Abstract] OR "ischaemic stroke"[Title/Abstract] OR "ischaemic strokes"[Title/Abstract] OR "noncardioembolic stroke"[Title/Abstract] OR "non-cardioembolic stroke"[Title/Abstract] OR "noncardioembolic ischemic stroke"[Title/Abstract] OR "non-cardioembolic ischemic stroke"[Title/Abstract] OR "cerebral infarction"[Title/Abstract] OR "brain infarction"[Title/Abstract] OR "transient ischemic attack"[Title/Abstract] OR "transient ischaemic attack"[Title/Abstract] OR TIA[Title/Abstract]) |
|  | **Intervention: Factor XI/XIa inhibitors** | **AND** ("Factor XI"[MeSH Terms] OR "factor XI inhibitor"[Title/Abstract] OR "factor XI inhibitors"[Title/Abstract] OR "factor XIa inhibitor"[Title/Abstract] OR "factor XIa inhibitors"[Title/Abstract] OR "activated factor XI"[Title/Abstract] OR "coagulation factor XIa"[Title/Abstract] OR FXI[Title/Abstract] OR FXIa[Title/Abstract] OR asundexian[Title/Abstract] OR milvexian[Title/Abstract] OR "BAY 2433334"[Title/Abstract] OR BAY2433334[Title/Abstract] OR "BMS-986177"[Title/Abstract] OR "BMS 986177"[Title/Abstract]) |
| **Embase.com (Elsevier)** | **Population: Stroke or transient ischemic attack** | ('cerebrovascular accident'/exp OR 'brain ischemia'/exp OR 'brain infarction'/exp OR 'transient ischemic attack'/exp OR stroke:ti,ab,kw OR strokes:ti,ab,kw OR 'ischemic stroke':ti,ab,kw OR 'ischemic strokes':ti,ab,kw OR 'ischaemic stroke':ti,ab,kw OR 'ischaemic strokes':ti,ab,kw OR 'noncardioembolic stroke':ti,ab,kw OR 'non-cardioembolic stroke':ti,ab,kw OR 'noncardioembolic ischemic stroke':ti,ab,kw OR 'non-cardioembolic ischemic stroke':ti,ab,kw OR 'cerebral infarction':ti,ab,kw OR 'brain infarction':ti,ab,kw OR 'transient ischemic attack':ti,ab,kw OR 'transient ischaemic attack':ti,ab,kw OR TIA:ti,ab,kw) |
|  | **Intervention: Factor XI/XIa inhibitors** | **AND** ('blood clotting factor 11'/exp OR 'blood clotting factor 11a'/exp OR 'asundexian'/exp OR 'milvexian'/exp OR 'factor XI inhibitor':ti,ab,kw OR 'factor XI inhibitors':ti,ab,kw OR 'factor XIa inhibitor':ti,ab,kw OR 'factor XIa inhibitors':ti,ab,kw OR 'activated factor XI':ti,ab,kw OR 'coagulation factor XIa':ti,ab,kw OR FXI:ti,ab,kw OR FXIa:ti,ab,kw OR asundexian:ti,ab,kw OR milvexian:ti,ab,kw OR 'BAY 2433334':ti,ab,kw OR BAY2433334:ti,ab,kw OR 'BMS-986177':ti,ab,kw OR 'BMS 986177':ti,ab,kw) |
| **Cochrane Library/CENTRAL** | **Population: Stroke or transient ischemic attack** | (stroke OR strokes OR "ischemic stroke" OR "ischemic strokes" OR "ischaemic stroke" OR "ischaemic strokes" OR "noncardioembolic stroke" OR "non-cardioembolic stroke" OR "noncardioembolic ischemic stroke" OR "non-cardioembolic ischemic stroke" OR "cerebral infarction" OR "brain infarction" OR "transient ischemic attack" OR "transient ischaemic attack" OR TIA):ti,ab,kw |
|  | **Intervention: Factor XI/XIa inhibitors** | **AND** ("factor XI inhibitor" OR "factor XI inhibitors" OR "factor XIa inhibitor" OR "factor XIa inhibitors" OR "activated factor XI" OR "coagulation factor XIa" OR FXI OR FXIa OR asundexian OR milvexian OR "BAY 2433334" OR BAY2433334 OR "BMS-986177" OR "BMS 986177"):ti,ab,kw |
| **ClinicalTrials.gov** | **Population: Stroke or transient ischemic attack** | (stroke OR strokes OR "ischemic stroke" OR "ischemic strokes" OR "ischaemic stroke" OR "ischaemic strokes" OR "noncardioembolic stroke" OR "non-cardioembolic stroke" OR "noncardioembolic ischemic stroke" OR "non-cardioembolic ischemic stroke" OR "cerebral infarction" OR "brain infarction" OR "transient ischemic attack" OR "transient ischaemic attack" OR TIA) |
|  | **Intervention: Factor XI/XIa inhibitors** | AND ("factor XI inhibitor" OR "factor XI inhibitors" OR "factor XIa inhibitor" OR "factor XIa inhibitors" OR "activated factor XI" OR "coagulation factor XIa" OR FXI OR FXIa OR asundexian OR milvexian OR "BAY 2433334" OR BAY2433334 OR "BMS-986177" OR "BMS 986177") |
|  | **Filters/settings** | Study type: Interventional studies; recruitment status: all; phase: all; age: adult/older adult where applicable; no language restriction; searched through May 2026. |

**Supplementary Table S1A.** Complete electronic search strategies used for database and trial-registry searches

| **Source** | **Platform/interface** | **Date searched** | **Coverage** | **Complete search strategy as executed** | **Restrictions/filters** | **Records retrieved** |
| --- | --- | --- | --- | --- | --- | --- |
| PubMed/MEDLINE | PubMed (National Library of Medicine) | May 2026 | Inception–May 2026 | Given in supplementary table 1A | None; no language or date restriction | 237 |
| Embase | Embase.com (Elsevier) | May 2026 | Inception–May 2026 | Given in supplementary table 1A | None | 746 |
| CENTRAL | Cochrane Library | May 2026 | Inception–May 2026 | Given in supplementary table 1A | None | 90 |
| ClinicalTrials.gov | ClinicalTrials.gov | May 2026 | Inception–May 2026 | Given in supplementary table 1A | None | 12 |

**Supplementary Table S1B.** PRISMA-S reporting details for electronic information sources

| **Variables** | **PACIFIC-Stroke (Shoamanesh 2022)** | | **AXIOMATIC-SSP (Sharma 2024)** | | **OCEANIC-Stroke (Sharma 2026)** | |
| --- | --- | --- | --- | --- | --- | --- |
|  | **Intervention (N=447)** | **Control (N=456)** | **Intervention (N=318)** | **Control (N=691)** | **Intervention (N=6162)** | **Control (N=6165)** |
| **Characteristics** | **Asundexian (50 mg only)** | **Placebo** | **Milvexian (25 mg twice daily)** | **Placebo** | **Asundexian (50 mg)** | **Placebo** |
| Age, yr, mean (SD) | 67·0 (10·0) | 66·6 (10·1) | - | - | 67.7 (10.8) | 67.5 (10.9) |
| Age, yr, median (IQR) | - | - | 72 (62-79) | 70 (62-77) | - | - |
| <65 | - | - | 97 (31%) | 208 (30%) | 2313 (37.5%) | 2308 (37.4%) |
| ≥65 to <75 | - | - | 97 (31%) | 255 (37%) | 2318 (37.6%) | 2318 (37.6%) |
| ≥75 | - | - | 124 (39%) | 228 (33%) | 1531 (24.8%) | 1539 (25%) |
| **Gender, (%)** |  |  |  |  |  |  |
| Male | 293 (66%) | 306 (67%) | 200 (63%) | 437 (63%) | 4099 (66.5%) | 4118 (66.8%) |
| Female | 154 (34%) | 150 (33%) | 118 (37%) | 254 (37%) | 2063 (33.5%) | 2047 (33.2%) |
| Race |  |  |  |  |  |  |
| White | 367 (82%) | 380 (83%) | 246 (77%) | 549 (79%) | 4105 (66.6%) | 4078 (66.1%) |
| Black | 8 (2%) | 3 (1%) | 6 (2%) | 15 (2%) | 143 (2.3%) | 139 (2.3%) |
| Asian | 68 (15%) | 66 (14%) | 63 (20%) | 122 (18%) | 1721 (27.9%) | 1742 (28.3%) |
| Other | 1 (<1%) | 1 (<1%) | 3 (1%) | 5 (1%) | 193 (3.1%) | 206 (3.3%) |
| **Geographical region** |  |  |  |  |  |  |
| North America | 5 (1%) | 6 (1%) | - | - | 783 (12.7%) | 782 (12.7%) |
| South America | - | - | - | - | 281 (4.6%) | 286 (4.6%) |
| Australia, Europe, and Israel | 248 (55%) | 255 (56%) | - | - | 3338 (54.2%) | 3329 (54.0%) |
| Eastern Europe | 131 (29%) | 131 (29%) | - | - | - | - |
| Asia/ Asia-Pacific | 63 (14%) | 64 (14%) | - | - | 1760 (28.6%) | 1768 (28.7%) |
| **Qualifying/Index event — no. (%)** |  |  |  |  |  |  |
| Ischemic stroke/Stroke | 447 (100%) | 456 (100%) | 240 (75%) | 526 (76%) | 5839 (94.8%) | 5838 (94.7%) |
| TIA/ High risk TIA | 0 | 0 | 77 (24%) | 163 (24%) | 323 (5.2%) | 325 (5.3%) |
| **Previous medical conditions** |  |  |  |  |  |  |
| Hypertension (n/%) | 347 (78%) | 346 (76%) | 240 (75%) | 549 (79%) | 4937(80%) | 4868(79%) |
| Diabetes (n/%) | 115 (26%) | 127 (28%) | 102 (32%) | 222 (32%) | 2134(34.6%) | 2115(34.3%) |
| Hypercholesterolemia/Hyperlipidemia | 256 (57%) | 246 (54%) | 176 (55%) | 398 (58%) | - | - |
| Heart Failure | 26 (6%) | 24 (5%) | - | - | - | - |
| Coronary artery disease | 46 (10%) | 37 (8%) | 34 (11%) | 84 (12%) | - | - |
| Myocardial infarction | 23 (5%) | 17 (4%) | - | - | - | - |
| Percutaneous coronary intervention or angioplasty, or coronary artery bypass grafting | 22 (5%) | 19 (4%) | - | - | - | - |
| Carotid endarterectomy or stenting | 4 (1%) | 2 (<1%) | - | - | - | - |
| Peripheral artery disease | 12 (3%) | 13 (3%) | - | - | - | - |
| Chronic Kidney Disease | 16 (4%) | 29 (6%) | - | - | - | - |
| Previous stroke or transient ischemic attack (Both) | 65 (15%) | 75 (16%) | - | - | 1310(21.2%) | 1345(21.8%) |
| Previous stroke | - | - | 52 (16%) | 91 (13%) | - | - |
| Previous transient ischemic attack | - | - | 31 (10%) | 60 (9%) | - | - |
| History of gastrointestinal bleeding | 6 (1%) | 7 (2%) | - | - | - | - |
| Pulmonary artery disease | N/R | N/R | 17 (5%) | 42 (6%) | - | - |
| Smoking/Tobacco use | 122 (27%) | 126 (28%) | 149 (47%) | 374 (54%) | - | - |
| Bodyweight (kg) | 79·2(16·5) | 79·2 (16·0) | - | - | - | - |
| **Qualifying Stroke Subtype — no./total no. (%)** |  |  |  |  |  |  |
| Large artery atherosclerosis | 89 (20%) | 76 (17%) | - | - | 2512/5839 (43.0) | 2484/5838 (42.5) |
| Small-vessel occlusion | 206 (46%) | 210 (46%) | - | - | 1290/5839 (22.1) | 1349/5838 (23.1) |
| Stroke of other determined aetiology/cause | 14 (3%) | 6 (1%) | - | - | 161/5839 (2.8) | 188/5838 (3.2) |
| Stroke of undetermined aetiology/cause | 137 (31%) | 162 (36%) | - | - | 1786/5839 (30.6) | 1710/5838 (29.3) |
| Cardioembolism | 1 (<1%) | 2 (<1%) | - | - | 89/5839 (1.5) | 107/5838 (1.8) |
| **Eligibility criterion, no. (%)** |  |  |  |  |  |  |
| Medical history of atherosclerosis | - | - | - | - | 1990(32.3%) | 2000(32.45%) |
| Extracranial or intracranial atherosclerosis proximal to the qualifying stroke | 154 (34%) | 142 (31%) | - | - | - | - |
| Carotid artery atherosclerosis identified by vascular imaging | 152 (34%) | 166 (36%) | - | - | - | - |
| Brain imaging demonstrating an acute non-lacunar infarct | N/R | N/R | - | - | 2989(48.5%) | 3007(48.8%) |
| **NIHSS score at randomization (IQR)** |  |  |  |  |  |  |
| Mean (SD) | 3·0 (2·3) | 2·9 (2·2) | - | - | - | - |
| 8-15 score | 16 (4%) | 17 (4%) | - | - | - | - |
| Median | - | - | 2 (0–3) | 2 (1–3) | 2.0 (1.0–4.0) | 2.0 (1.0–4.0) |
| ≤3 | - | - | - | - | 4087(70.0%) | 4079(69.9%) |
| 4-7 | - | - | - | - | 1385(23.7%) | 1375(23.6%) |
| ≥8 | - | - | - | - | 365(6.3%) | 382(6.5%) |
| Day 7 mRS score | 1·0 (1·0–2·0) | 1·0 (1·0–2·0) | - | - | - | - |
| Dual antiplatelet therapy before index event | 8 (2%) | 8 (2%) | - | - | - | - |
| Dual antiplatelet therapy after index event | 192 (43%) | 199 (44%) | - | - | - | - |
| Planned dual antiplatelet therapy with aspirin and P2Y12 inhibitor at randomization — no. (%) | - | - | - | - | 3859 (62.6%) | 3853 (62.5%) |
| Intravenous thrombolysis, endovascular therapy, or both — no./total no. (%) | - | - | - | - | 1608/5839 (27.5) | 1593/5838 (27.3) |
| Thrombolysis/Intravenous thrombolysis only | - | - | - | - | 1146/5839 (19.6) | 1168/5838 (20.0) |
| Endovascular thrombectomy/therapy only | - | - | - | - | 202/5839 (3.5) | 169/5838 (2.9) |
| Blood Pressure at randomization, mmHg | 139 (14) / 79 (10) | 137 (14) / 79 (10) | - | - | - | - |
| Systolic BP, mmHg, median (IQR) | - | - | 146 (131-160) | 145 (132-160) | - | - |
| Diastolic BP, mmHg, median (IQR) | - | - | 80 (73-90) | 80 (72-89) | - | - |
| eGFR, mL/min per 1·73 m²§ | 78 (20) | 79 (22) | - | - | - | - |
| eGFR, mL/min per 1·73, median (IQR) | - | - | 84 (68–98) | 83 (69–99) | - | - |
| **Time from event to randomization (hrs)** |  |  |  |  |  |  |
| ≤24 h | 51 (11%) | 49 (11%) | - | - | 335(5.4%) | 356(5.7%) |
| >24 to ≤48 h | - | - | - | - | 2190(35.5%) | 2150(34.8%) |
| >48 to ≤72 h | - | - | - | - | 3592(58.3%) | 3630(58.9%) |
| >72 h | - | - | - | - | 45(0.7%) | 29(0.5%) |
| **Median time from symptom onset to first dose (h)** |  |  |  |  |  |  |
| <12 | - | - | 15 (5%) | 21 (3%) | - | - |
| 12–24 | - | - | 38 (12%) | 38 (12%) | - | - |
| >24–48 | - | - | 244 (77%) | 539 (78%) | - | - |
| >48 | - | - | 14 (4%) | 21 (3%) | - | - |
| Not reported | - | - | 7 (2%) | 11 (2%) | - | - |
| tPA, EVT, or both | - | - | 20 (6%) | 81 (12%) | - | - |

**Supplementary Table S2.** Baseline Characteristics of the included randomized clinical trials

| **Meta-analysis outcome** | **PACIFIC-Stroke** | **AXIOMATIC-SSP** | **OCEANIC-STROKE** | **Harmonization used in meta-analysis** | **Important limitation** |
| --- | --- | --- | --- | --- | --- |
| **Major bleeding** | ISTH major bleeding | ISTH major bleeding available from supplementary analysis | ISTH major bleeding | ISTH major bleeding pooled across all three trials | Good comparability |
| **Major or clinically relevant non-major bleeding** | ISTH major or CRNM bleeding | ISTH major or CRNM bleeding | ISTH major or CRNM bleeding | Trial-reported ISTH composite pooled | AXIOMATIC definition included bleeding associated with treatment-emergent adverse events and asymptomatic ICH detected on day-90 MRI |
| **Clinically relevant non-major bleeding** | ISTH CRNM bleeding | ISTH CRNM bleeding | ISTH CRNM bleeding | Trial-reported CRNM bleeding pooled | Some differences in ascertainment/reporting |
| **Minor/non-actionable bleeding** | Trial-defined minor bleeding | BARC type 1 non-actionable bleeding | Trial-defined minor bleeding | BARC type 1 in AXIOMATIC treated as closest corresponding category | Definitions not identical |
| **Intracranial/intracerebral hemorrhage** | Primary intracerebral hemorrhage | Modified BARC type 3c intracranial hemorrhage, including symptomatic hemorrhagic transformation | Symptomatic intracranial hemorrhage, including intracerebral hemorrhage | Closest clinically corresponding serious intracranial bleeding category pooled | Important definitional heterogeneity |
| **Fatal bleeding** | Not separately extractable / not reported for selected comparison, if applicable | No fatal bleeding in either selected group | Fatal bleeding | Pooled when estimable; zero-zero study contributed no statistical weight | Very few events |

**Supplementary Table S3:** Trial-specific definitions and harmonization of safety outcomes

**Abbreviations:** BARC, Bleeding Academic Research Consortium; CRNM, clinically relevant non-major; ICH, intracranial hemorrhage; ISTH, International Society on Thrombosis and Haemostasis; MRI, magnetic resonance imaging.
